# Correction: On-surface synthesis of organometallic nanorings linked by unconventional intermediates of the Ullmann reaction

**DOI:** 10.1039/d5sc90129d

**Published:** 2025-06-09

**Authors:** Xiaoyang Zhao, Liqian Liu, Zhipeng Zhang, Tianchen Qin, Jun Hu, Lei Ying, Junfa Zhu, Tao Wang, Xinrui Miao

**Affiliations:** a State Key Laboratory of Luminescent Materials and Devices, College of Materials Science and Engineering, South China University of Technology Guangzhou 510640 P. R. China msxrmiao@scut.edu.cn; b National Synchrotron Radiation Laboratory, University of Science and Technology of China Hefei 230029 P. R. China; c State Key Laboratory of Organometallic Chemistry, Shanghai Institute of Organic Chemistry, Chinese Academy of Sciences Shanghai 200032 P. R. China taowang@sioc.ac.cn

## Abstract

Correction for ‘On-surface synthesis of organometallic nanorings linked by unconventional intermediates of the Ullmann reaction’ by Xiaoyang Zhao *et al.*, *Chem. Sci.*, 2025, **16**, 9348–9356, https://doi.org/10.1039/D5SC01269D.

The authors regret that there was an error in the sentence in lines 6–8 in the left column on page 9352 of the original article.

The text originally read: “A 6-membered nanoring containing two C–Cu–C and four C–Cu–Br–Cu–C bonds is shown in Fig. 2f and g.”

The sentence should read: “A 6-membered nanoring containing four C–Cu–C and two C–Cu–Br–Cu–C bonds is shown in Fig. 2f and g.”

The Royal Society of Chemistry apologises for these errors and any consequent inconvenience to authors and readers.

